# 

**Published:** 1898-08

**Authors:** 


### U. S. V. M. A. MEETING, 1898.

### OMAHA, SEPTEMBER 6, 7, 8.

*Some Points to be Specially Remembered.*

THE Executive Committee meets on the afternoon of the 5th, and you should have your application for membership before it at that time.

Your attendance will demonstrate your interest in the adoption of surgical clinics as a feature of our meetings.

Modes of control, methods of local and general anæsthesia, are of everyday importance to you.

How others do certain operations is sure to afford you some new ideas and points and, perhaps, bring out some valuable suggestion from you. All the operations are such as you may be called to do any day, and you want to know the best methods extant.

Meat-inspection, of so much vital importance to all, may not be a pressing question in your territory to-day, but can you say it will not be to-morrow? You may not care for such work yourself, but this does not lessen your need of knowing all about it. You have colleagues in the profession, fellow-members in the Association specially equipped for this work, and your familiarity with the subject and appreciation of its importance may aid them, and you do your colleagues and community a well-deserved favor in this direction.

The specimens shown will be an object-lesson of the greatest importance, and Omaha, as one of the largest packing centres of the world, affords an excellent opportunity of studying a practical aspect of the question.

Rabies and osteoporosis are to-day two of the most important diseases extant. On the one hand, to know how to diagnose accurately and how best to deal with these cases when they appear, and render the best services to mankind, are important problems. On the other, the import of osteoporosis from the commercial side, and how best it may be controlled and limited, is an everyday demand in a goodly proportion of our members. What it is, and how best to control its inroads, are your and my field to open and solve. Your experience may contain valuable points.

The Exposition in all its grandeur is there to attract you, interest and educate you, and it will afford the last opportunity this century now closing of seeing the beautiful sights with which the Trans-Mississippi Exhibition abounds. Take your wife or parents with you, and let them see and know about these undertakings in the Central West.

There are questions to be determined of the greatest import to all concerned, and your views in these matters will best help to solve them in the interest of the greater number.

Messrs. Charles F. Squibb and W. W. Dixon, of Brooklyn, representatives of the well-known firm of E. R. Squibb & Sons, will accompany the JOURNAL " special " to Omaha on September 3d.

President Jobson, of the Pennsylvania State Veterinary Medical Association, has appointed Drs. J. C. Foelker, of Allentown, and F. F. Hoffman, of Brookville, and J. Curtis Michener, of Colmar, delegates to the U. S. V. M. A.

Treasurer William H. Lowe, of Paterson, N. J., will be on the JOURNAL " special " for Omaha.

The Minnesota and Manitoba delegations will leave Minneapolis at 7.10 P.M., St. Paul at 7.45 P.M., September 5th, over the Omaha route.

Dr. Leonard Pearson, State Veterinarian of Pennsylvania, will join the "JOURNAL Special" at Harrisburg.

President Salmon, of Washington, and State Veterinarian Clement journey by the "JOURNAL Special."

Dr. J. C. Foelker, of Allentown, State Delegate from the Pennsylvania Association, will be on the "JOURNAL Special."

Prof. John W. Adams, of the Veterinary Department of the University of Pennsylvania, will include a trip to his old home at St. Paul, Minn. He also travels by the "JOURNAL Special."

Drs. B. M. Underhill, of Media, and W. P. Phipps, of Lionville, Pa., will be in attendance at Omaha. They are among the "JOURNAL Special" passengers.

All veterinarians in the Eastern and central Western States may join readily the "JOURNAL Special" at Chicago, leaving the latter city over the Burlington route on Sunday evening at 5.45 P.M.

Mr. and Mrs. William P. Allen, of Philadelphia, who were visitors to the World's Fair Congress of our Association, will be visitors to the Omaha meeting; they go by the "JOURNAL Special."

Editor Bell, of the *American Veterinary Review*, will be numbered among the "JOURNAL Special" passengers.

With the Treasurer, Dr. Wm. H. Lowe, of Paterson, N. J., among those who make up the Eastern delegation of the "JOURNAL Special," the commissary ought to well provided for. It is always safe to be in close touch with the man " who sits on the chist."

### PERSONALS.

Dr. William H. Kelly, of Albany, N. Y., expects to join the JOURNAL special at Chicago.

President D. E. Salmon, of Washington, D. C., will join the JOURNAL special at Harrisburg, Pa.

Mr. Harold Sorby, of the Pasteur Vaccine Company, will join the JOURNAL special over the Burlington Route from Chicago to Omaha.

Dr. Frank P. Shannon, Assistant Inspector at Fort Worth, Tex., has been transferred to Los Angeles, Cal. Dr. John A. Kiernan, of Chicago, to Fort Worth, Tex. Dr. Frank C. Eels, of Brownsville, Tex., to the National Stock Yards, Chicago, Ill. Dr. James Keely has been sent to Brownsville, Tex.

Dr. M. E. Conard's (West Grove, Pa.) paper on "What our Dairy Cows Inherit," was published in a July number of *The Jersey Bulletin*, and criticised by the editor of that breezy publication.

Dr. H. Bowen, of Mount Gilead, O., is at present deputy sheriff of Morrow County.

Messrs. Waugh, Jennings, and Kingan, of Pittsburg, will be more alert in the future as to calls by a special messenger who has come to town and finds himself unexpectedly short in funds and secures temporary loan.

Dr. A. W. Bitting, of Purdue University, Ind., is out in a bulletin on the importance of car and transportation as well as pen disinfection in livestock shows, especially to lessen the danger of transmission of hog-cholera and swine-plague.

Dr. John W. Adams spent a portion of July quietly resting at Warsaw, N. Y., accompanied by his family.

Dr. James W. Sallade, of Pottsville, was a professional visitor to York, Pa., in July.

Dr. John E. Spindler, graduate of the Veterinary Department of the University of Pennsylvania, class of 1898, has located at Tyrone, Pa.

Mr. R. A. Pearson, of the Dairy Division, Department of Agriculture, was a visitor to Philadelphia for a week in July.

Dr. Ray J. Stanclift, who recently graduated at the New York State Veterinary College, has returned to Americus, Ga., to practice.

Dr. W. E. A. Wyman has resigned his position as Professor of Veterinary Science at the Clemson Agricultural College, S. C., and entered upon private practice at Milwaukee, Wis.

Dr. A. G. Van Tine, of Millgrove, has returned to Clarence, N. Y.

Dr. George W. Turner, formerly of Highwood, Ill., now with the United States Army at camp George H. Thomas, Chickamauga, Ga., is called "Chief Veterinarian of the Camp," a recognized honor, not by commission.

Dr. Leonard Pearson included in his foreign trip a visit to Berlin and Bremen, Germany. While in Paris he was for a time the guest of Prof. A. Liautard, who now makes his home in "Sunny France."

M. A. Francis, State Veterinarian of Texas, was a visitor to Ohio in August, and contemplated extending his visit to the East.

The Rochester, N. Y., branch of the Horseshoers' National Protective Union issued some five diplomas of proficiency to as many young shoers in August. Dr. J. C. McKenzie, a member of the U. S. V. M. A., is the active spirit of this branch of the organization, and as instructors in the school of farriery conducted there he was favored with the assistance of Drs. Leroy M. Webber and A. Drinkwater.

Dr. A. S. Alexander, of Evanston, Ill., was a contributor the August 3d number of the *Breeders' Gazette* on the "Care of Unshod Feet."

Dr. M. E. Knowles, of Helena, Mont., takes a keen interest in the breeding and development of trotting horses.

Veterinarians William H. Kelly, of Albany, and V. A. Moore, of Ithaca, N. Y., have been investigating for the State reported outbreaks of symptomatic anthrax, tuberculosis, and hog-cholera at Middleville, Jordanville, and Saratoga, respectively.

Dr. T. J. Shinkwin, graduate of Harvard Veterinary Department, has recently been assisting Dr. M. O'Connell, of Holyoke, during the latter's illness.

Dr. Harry Walters, of Wilkesbarre, Pa., was a sojourner at Manhattan Beach for a short period early in July, and a visitor to Philadelphia.

Dr. F. K. Nice, of Philadelphia, has become the proprietor of the Tioga Veterinary Hospital.

Dr. L. Van Es, of Mobile, Ala., conducts a veterinary hospital in connection with his practice.

Dr. B. J. Senseman, of Philadelphia, was a sojourner at Atlantic City for a week in August, nursing an injured limb, the result of a kick from a mule.

Dr. C. L. Megowan holds the position of city milk- food-, and market-inspector for Sacramento, California.

Dr. John W. Adams, of Philadelphia, spent some two weeks in August in Western Pennsylvania on special work of the State Live Stock Sanitary Board.

Dr. T. E. Maloney, formerly of Boston, who has been in the Western quarantine service for some months past, has been transferred to his former place and home.

Dr. W. H. Mattson, of Camp Ground, Pa., examines all the cattle and dairies furnishing milk to the creamery producing the famed P. E. Sharpless butter.

Dr. George R. Jobson, of Franklin, Pa., was recently appointed to the meat-inspection force of the Bureau of Animal Industry and assigned to duty at Chicago.

Dr. C. W. Boyd, of Allegheny, Pa., has been resting at the famous seaside resort, Atlantic City, N. J., for a period.

Dr. William Jobson, of Franklin, Pa., having taken the degree of M.D. at the Medical Department of the Columbian University, has recently accepted the position of physical director of the Young Men's Christian Association Gymnasium at Franklin.

Dr. Edward M. Ranck has removed from 4025 Market Street to 427 North Forty-first Street, and A. W. Ormiston from 19 Levering Street, Mt. Airy, to 6219 Germantown Avenue, Philadelphia.

Dr. J. P. Turner, recently of the United States Inspection Service at St. Louis, Mo., has received the appointment of Dairy Inspector for the District of Columbia. He succeeds Dr. C. Barnwell Robinson, who has filled this position for the past three years.

Dr. A. W. Bitting's paper on "A New Photo-micrographic Apparatus," presented to the Indianapolis Academy of Science, has appeared in bulletin form.

Veterinarian James Jefferson Reitz, of Pennsylvania, and Eckley Storrs, of Willimantic, Conn., are students of the second year at the Jefferson Medical College of Philadelphia. Dr. Alexander Glass, of Philadelphia, is among the special students.

Dr. W. R. Smith, graduate of Veterinary Department of Harvard University, class of 1898, has located at West Brookfield, Mass.

Dr. Cooper Curtice, of Moravia, N. Y., has recently been engaged in special work for the New York State Board of Health at the Buffalo Stock Yards.

Lieutenant-Colonel R. S. Huidekoper, one of the editors of the Journal, sailed for Porto Rico from Newport News, Va., on July 26th, as Chief-Surgeon of the First Army Corps.

Dr. W. F. Smith, of Amsterdam, N. Y., fills the rôle of veterinarian to the Hurricana Stud Farm.

Dr. J. C. McNeill, of Pittsburg, Pa., was a visitor to Philadelphia in July and a sojourner at Atlantic City for a short stay.

Dr. A. L. Baum, of Telford, Pa., was a visitor at the Veterinary Department of the Univercity of Pennsylvania in July.

